# Importance of Toxin A, Toxin B, and CDT in Virulence of an Epidemic *Clostridium difficile* Strain

**DOI:** 10.1093/infdis/jit426

**Published:** 2013-08-08

**Authors:** Sarah A. Kuehne, Mark M. Collery, Michelle L. Kelly, Stephen T. Cartman, Alan Cockayne, Nigel P. Minton

**Affiliations:** Clostridia Research Group, Nottingham Digestive Diseases Centre, NIHR Biomedical Research Unit, School of Life Sciences, University of Nottingham, United Kingdom

**Keywords:** *Clostridium difficile* infection, TcdA, TcdB, pathogenesis, CDT

## Abstract

*Clostridium difficile* infection is the main cause of healthcare-acquired diarrhea in the developed world. In addition to the main virulence factors toxin A and B, epidemic, PCR Ribotype 027 strains, such as R20291, produce a third toxin, CDT. To develop effective medical countermeasures, it is important to understand the importance of each toxin. Accordingly, we created all possible combinations of isogenic toxin mutants of R20291 and assessed their virulence. We demonstrated that either toxin A or toxin B alone can cause fulminant disease in the hamster infection model and present tantalizing data that *C. difficile* toxin may also contribute to virulence.

**(See the editorial commentary by Young and Hanna on pages 9–11.)**

Two large cytotoxins, toxins A and B, remain the only definitive virulence factors of *Clostridium difficile.* Their relative importance in *C. difficile* infection (CDI), however, remains a subject of debate. Early studies were hampered by a dearth of available genetic tools with which the stable isogenic mutants required for comparative virulence studies could be created. This obstacle was removed by the development, and later refinement, of ClosTron technology [1].

To date, 2 independent studies have investigated the relative role of toxins A and B in virulence through the creation of isogenic toxin mutants and their analysis in the hamster infection model. With respect to toxin A, conflicting results were obtained: one study demonstrated that toxin A alone could not cause disease in hamsters, whereas the other showed that an equivalent mutant in the same animal model was virulent [2, 3]. In both cases, the mutants were generated in erythromycin-susceptible derivatives of strains 630. Because these strains were independently isolated following repeated serial passage in antibiotic-free medium, the emergence of ancillary mutations that could have influenced virulence cannot be discounted.

It has been suggested that this apparent paradox could be resolved by the examination of other strains [4], particularly polymerase chain reaction (PCR) ribotype 027 epidemic strains (also referred to as 027/NAP1/B1 strains), that are associated with more severe disease, higher relapse rates, increased mortality, and greater resistance to fluoroquinolone antibiotics [5]. PCR ribotype 027 strains additionally produce a binary toxin, called *C. difficile* toxin (CDT). A role for CDT in virulence has yet to be established. It shares similarity with *Clostridium perfringens* iota toxin and has an adenosine diphosphate–ribosyltransferase activity that covalently modifies cell actin. CDT, however, shows no cytotoxicity toward Vero cells without prior trypsinization and has no adverse effects when purified and injected into mice. [6] Yet, CDT protein levels were shown to be much higher when measured in situ than when tested in vitro [7]. By use of in vitro assays, it has additionally been shown that purified binary toxin mediates increased adherence of *C. difficile* to epithelial cells through the formation of protrusions [8]. This suggests a role for binary toxin in adherence and colonization. In the current investigation, we focused on the creation and testing of isogenic toxin mutants in the PCR ribotype 027 strain R20291, an isolate epidemic in the United Kingdom that was responsible for 2 outbreaks of CDI at the Stoke Mandeville hospital in 2003 and 2004.

## METHODS

### Strains and Growth Conditions

*Escherichia coli* strains used were TOP10 (Invitrogen) as a cloning host and *E. coli* CA434 [9] as a conjugal donor. *C. difficile* strains used were R20291 and mutants. *E. coli* cultures were aerobically grown with shaking on Luria Bertani medium at 37°C unless stated otherwise. *C. difficile* cultures were cultivated in BHIS [3] or TY [3] at 37°C in an anaerobic workstation (Don Whitley Scientific, Shipley, United Kingdom). Antibiotics used in this study are detailed in the Supplementary Materials.

### Molecular Biology Techniques

Qiagen mini prep kits were used to purify plasmids. Genomic DNA was obtained by phenol-chloroform extraction. Digests, PCRs, and DNA purification were undertaken according to general protocols [10]. DNA Sanger sequencing was performed by Source Biosciences (Nottingham, United Kingdom).

### Construction and Characterization of Mutants

The following *C. difficile* single-mutant strains were made from the parental strain R20291, using ClosTron technology as described elsewhere [11]: *tcdA*^−^, *tcdB*^+^, *cdtA*^+^ (A^−^B^+^C^+^); *tcdA*^+^, *tcdB*^−^, *cdtA*^+^ (A^+^B^−^C^+^); and *tcdA*^+^, *tcdB*^+^, *cdtA*^−^ (A^+^B^+^C^−^). The *tcdA*^−^, *tcdB*^−^, *cdtA*^+^ (A^−^B^−^C^+^) double-mutant strain was made from the A^+^B^−^C^+^ mutant, using a *catP*-based ClosTron and the pseudo-suicide vector principle [3]. The other 2 double mutants were made from the A^+^B^+^C^−^ mutant. The triple mutant was made from the A^−^B^−^C^+^ double mutant. Retargeted plasmids and primers used to verify insertions are listed in the Supplementary Materials.

### Southern and Western Blots

Southern and Western blots were performed as described previously [3].

### Resequencing of ClosTron Mutants

Genomic DNA of the created toxin mutants was isolated and subjected to Illumina sequencing (GATC). The obtained sequences were mapped to R20291 National Center for Biotechnology Information accession number NC_013316.1 and then analyzed for single-nucleotide polymorphisms (SNPs) and insertions and deletions (InDels), using CLC-bio.

### Cell Toxicity Assays

Cell cytotoxicity assays were performed as previously described [3].

### Hamster Infection Model

We used a block design with final group sizes of 8–10 animals. Female golden Syrian hamsters (weight, 100–130 g) were housed singly in individually ventilated cages. Each hamster received an oral dose of clindamycin (30 mg/kg) 7 days before being infected orally with 10^4^ *C. difficile* spores. Hamsters were monitored 3–4 times/day after infection and were assessed for several parameters, including presence and severity of diarrhea, weight loss, level of activity, starey coat, sunken eyes, hunched posture, and response to stimulus. A scoring system based on the severity of changes observed (ranging from 0 to 3 for each parameter) was used to quantify changes in the condition of the animals, which were euthanized when a predetermined cumulative value was reached. The hamsters were handled individually in a microbiological safety cabinet. In line with United Kingdom Home Office and local ethics review board requirements to reduce animal suffering, an alternative to death was used as the end point [3].

Fecal pellets were collected daily and plated to determine the presence of *C. difficile*. Cecum samples from each hamster were homogenized and plated, and *C. difficile* counts were determined. PCR was performed to determine the genotype of each strain recovered from hamsters (Supplementary Materials).

## RESULTS AND DISCUSSION

Throughout this study, the insertion of a group II intron, using ClosTron technology, into the targeted gene was confirmed by the nucleotide sequencing of DNA fragments, generated by PCR, that span the intron junctions. The absence of insertion at secondary sites was confirmed by Southern blot, and functional inactivation of the respective toxin genes was shown by appropriate Western blots (Supplementary Figure 1). The genomes of the created mutants were resequenced and shown to harbor no additional mutations (SNPs and InDels) to those intended.

**Figure 1.**
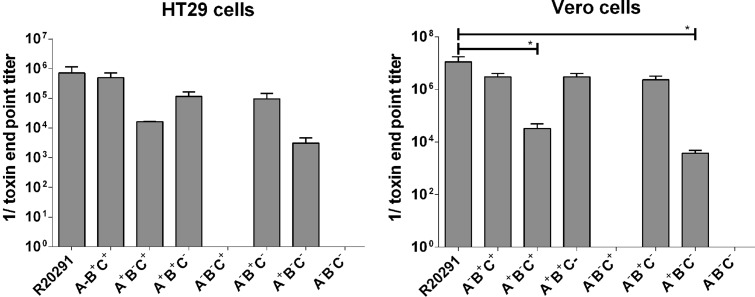
In vitro cytotoxicity. Supernatants of the parental strain R20291 and the 7 mutants, A^−^B^+^C^+^, A + B^−^C^+^, A^−^B^−^C^+^, A^−^B^+^C^−^, A^+^B^−^C^−^, and A^−^B^−^C^−^ (see Methods for definitions), were used in cell culture assays to measure cytotoxicity. HT29 cells (*A*) and Vero cells (*B*) were cultured to a flat monolayer before adding *Clostridium difficile* supernatants in 4-fold dilutions series. After a 24-hour incubation period, toxin end point titers were determined. Data represent mean values ± SD; n = 3. Statistical analyses were performed using 1-way analysis of variance, followed by Dunnett's multiple comparison test. *P < .05.

Strain R20291 produces CDT in addition to toxins A and B. As the primary goal of our study was to examine the relative roles of toxins A and B in CDI, we initially undertook all our studies in strains in which the binary toxin had been inactivated by a ClosTron-derived insertion in the *cdtA* gene encoding for the catalytic domain of the toxin. Hereafter, toxin A will be referred to as “A,” toxin B as “B,” and CDT as “C.” A “+” indicates that the gene is still intact and, hence, that the toxin is produced, whereas a “−” indicates that the respective gene has been interrupted and that the corresponding toxin is no longer produced. Accordingly, mutants were generated producing either toxin A (A^+^B^−^C^−^) or toxin B (A^−^B^+^C^−^) alone or neither (A^−^B^−^C^−^). The toxicity of the 3 mutant strains created was initially measured using a cytotoxicity assay on HT29 (human colon carcinoma) cells and Vero (African green monkey kidney) cells. The strain only producing toxin B (A^−^B^+^C^−^) still displayed a level of cytotoxicity similar to that of the wild-type strain (A^+^B^+^C^+^), whereas the strain only expressing toxin A (A^+^B^−^C^−^) showed attenuated toxicity (Vero cell assay, *P* < .05) (Figure 1). The triple mutant (A^−^B^−^C^−^) displayed no cytotoxicity. Thereafter, the in vivo virulence of all 3 mutants was compared to that of the wild-type strain in the hamster infection model. A total of 8–10 female golden Syrian hamsters were infected orally with 10^4^ spores of the respective strains and monitored for signs of CDI. Changes in condition were regularly scored [3], and the experiment was terminated when hamsters developed fulminant CDI, as described by Kuehne et al [3]. The experiment duration was 14 days.

**Figure 2.**
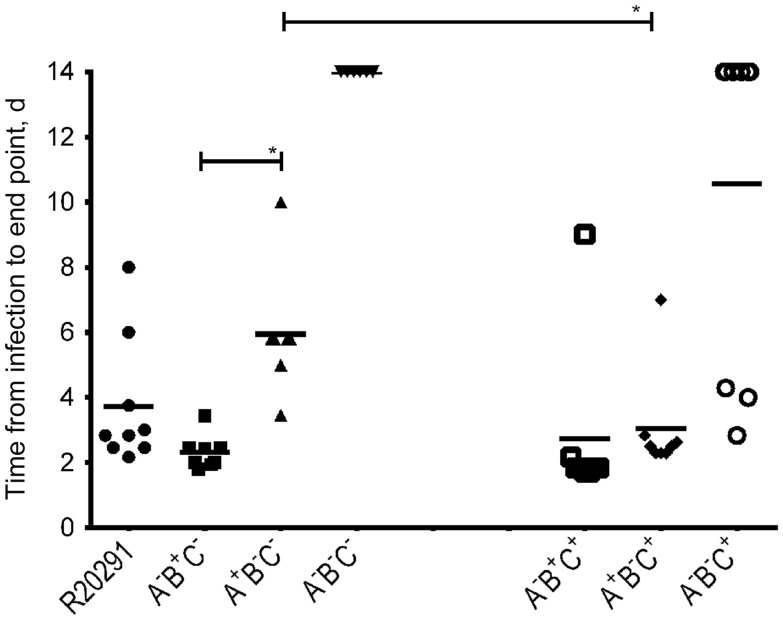
Virulence of *Clostridium difficile* strains in hamsters. Groups of 8–10 hamsters were challenged with *C. difficile* R20291 (A^+^B^+^C^+^) or one of the toxin mutant strains, A^−^B^+^C^−^, A^+^B^−^C^−^, A^−^B^−^C^−^, A^−^B^+^C^+^, A + B^−^C^+^, or A^−^B^−^C^+^ (see Methods for definitions). The time from infection to end point is presented in days. The duration of the experiment was set at 14 days. Statistical analyses were performed using 1-way analysis of variance and the Student *t* test. *P < .05.

As expected, all hamsters that were colonized with the wild-type R20291 strain (9 of 10 infected were colonized) succumbed to the disease in a mean time (±SD) of 3.7 ± 1.97 days after infection (Figure 2). All 8 hamsters infected and colonized with the triple toxin mutant strain (A^−^B^−^C^−^) survived for the duration of the experiment and showed no signs or symptoms of CDI. In contrast, all of the hamsters (8 of 8) that were infected and then colonized with a strain making only toxin B (A^−^B^+^C^−^) succumbed to CDI, with a mean time (±SD) from infection to end point of 2.3 ± 0.52 days. Crucially, all hamsters (7 of 9) infected and then colonized with a strain only making toxin A (A^+^B^−^C^−^) also developed clinical signs of CDI, with a mean interval (±SD) of 5.9 ± 1.98 days from infection to end point. These data are entirely consistent with the equivalent experiments conducted previously with mutants generated in strain 630Δ*erm*; that is to say, an isogenic strain producing toxin B alone is more virulent than an isogenic strain producing only toxin A (*P* < .05), but the latter is still able to cause disease in hamsters. Given the unknown role of CDT in disease, we created a further series of toxin A and B mutants in which the binary toxin genes were still functional. Unsurprisingly, the toxin A and B mutants with this C^+^ background (A^−^B^−^C^+^) were nontoxigenic under the experimental conditions used in vitro. The strains still expressing toxin B and CDT (A^−^B^+^C^+^) and those expressing toxin A and CDT (A^+^B^−^C^+^) showed high levels of cytotoxicity, although levels in the latter case were slightly less than those for the wild-type strain (Vero cell assay, *P* < .05). When tested in vivo*,* it was noted that all of the hamsters (8 of 8) infected and colonized with the single toxin A (A^−^B^+^C^+^) and toxin B mutants (A^+^B^−^C^+^) developed terminal CDI, with mean intervals (±SD) of 2.7 ± 2.53 days and 3.0 ± 1.61 days from infection to end point, respectively. Particularly striking was the apparently increased virulence (*P* < .05) of the A^+^B^−^C^+^ mutant (mean time from infection to end point, 3.0 days), compared with that of the A^+^B^−^C^−^ mutant (mean time from infection to end point, 5.9 days), suggesting that the presence of CDT may accentuate the virulence of a toxin B mutant strain producing toxin A alone. Thus, CDT may act in concert with toxin A to increase virulence.

It is worth noting that the hamsters infected with some single-mutant strains succumbed earlier, on average, to disease than hamsters infected with the wild-type strain. A likely explanation for this is the introduction of the *ermB* gene (through the mutagenesis procedure) into the chromosome of the mutants, which confers resistance to clindamycin. Because the wild-type strain is susceptible to clindamycin, the use of this antibiotic in the infection procedure may have affected the ability of this strain to colonize efficiently.

Intriguingly, in the case of the double toxin mutant (A^−^B^−^C^+^; ie, an isogenic mutant producing only CDT), 3 of 9 animals succumbed to disease (Figure 2). It should, however, be emphasized that these 3 animals did not show typical symptoms of CDI. Rather than producing loose feces and having a cecum with diffuse hemorrhage, they only showed signs of wet tail. Furthermore, we observed some hemorrhage and inflammation in their small intestines, which was not seen in any other animals. These observations are in keeping with a previous suggestion that *C. difficile* can cause infection of the small intestine [12]. Moreover, it has previously been shown that binary toxin can cause enterotoxic effects in the rabbit ileal loop assay [13]. This could point to a role of binary toxin in *C. difficile* virulence.

In conclusion, our studies reaffirm the previous finding that isogenic virulent strains of *C. difficile* producing toxin A alone cause disease in the hamster and reenforce our view that the development of diagnostic tests, vaccines, and therapeutics should focus on both toxin A and toxin B. Our experiments have also produced tantalizing evidence that CDT may contribute to disease. It has previously been suggested that the presence of binary toxin is linked to more severe disease outcomes [14], a view further supported by McEllistrem et al [15]. Moreover, the presence of CDT in all representatives of certain so-called hypervirulent strains (eg, PCR ribotype 027 and ribotype 078 strains) provides additional compelling evidence that it contributes to virulence. Further research will be necessary before the role of CDT in disease is fully understood, but in the meantime it may be prudent to test for its presence in clinical isolates through appropriate diagnostic tests.

## Supplementary Data

Supplementary materials are available at *The Journal of Infectious Diseases* online (http://jid.oxfordjournals.org/). Supplementary materials consist of data provided by the author that are published to benefit the reader. The posted materials are not copyedited. The contents of all supplementary data are the sole responsibility of the authors. Questions or messages regarding errors should be addressed to the author.

Supplementary Data
