# Supplementary material for: Importance of Toxin A, Toxin B, and CDT in Virulence of an Epidemic Clostridium difficile Strain
Source: J Infect Dis. 2013 Aug 8;209(1):83–6. doi: 10.1093/infdis/jit426 (PMC3864386; doi:10.1093/infdis/jit426)
Supplement: Supplementary Data [file supp_209_1_83__index.html]

The importance of toxin A, toxin B and CDT in virulence of an epidemic Clostridium difficile strain — Importance of Toxin A, Toxin B, and CDT in Virulence of an Epidemic Clostridium difficile Strain — Importance of Toxin A, Toxin B, and CDT in Virulence of an Epidemic Clostridium difficile Strain — Supplementary Data 

# Importance of Toxin A, Toxin B, and CDT in Virulence of an Epidemic *Clostridium difficile* Strain

## Supplementary Data

Supplementary Data

**Files in this Data Supplement:**

- Supplementary Data - Docx file
