# Supplementary material for: Importance of Toxin A, Toxin B, and CDT in Virulence of an Epidemic Clostridium difficile Strain
Source: J Infect Dis. 2013 Aug 8;209(1):83–6. doi: 10.1093/infdis/jit426 (PMC3864386; doi:10.1093/infdis/jit426)

**The importance of toxin A in virulence of an epidemic *Clostridium difficile* strain**

Sarah A. Kuehne, Mark M. Collery, Michelle L. Kelly, Stephen T. Cartman, Alan Cockayne & Nigel P. Minton

**Supplementary data**

**Methods**

**Antibiotics**

Antibiotics were used at the following concentrations where appropriate: chloramphenicol (25 µg/ml or 12.5 µg/ml), thiamphenicol (15 µg/ml), spectinomycin (250 µg/ml or 750 µg/ml), lincomycin (20 µg/ml), tetracycline (10 µg/ml), D-cycloserine (250 µg/ml) and cefoxitin (8 µg/ml).

**Retargeting plasmids and primers used to create and characterize the toxin mutants**

The following retargeted plasmids pMTL007C-E2::Cdi-*tcdA*-1584s, pMTL007C-E2::Cdi-*tcdB*-1511a and pMTL007C-E2::Cdi-*cdtA*-426s were used for the single mutants. The plasmids pMTL007S-C7::PBP1::Cdi-*tcdA*-1584s and pMTL007S-C7::PBP1::Cdi-*tcdB*-1511a were used to make the double mutants and pMTL007T-S7::PBP1::Cdi-*cdtA*-426s for the triple mutant. To verify the correct insertions, primers used for *tcdA* were: Cdi-tcdA-F2 and Cdi-tcdA-R2, for *tcdB* were: Cdi-tcdB-F1 and Cdi-tcdB-R1 [[1](#_ENREF_1)] and for *cdtA* were: Cdi-027-*cdtA*-F1 (5’- TCAAGAGTTAATTAAACTAATATTGGGAGG-3’) and Cdi-027-*cdtA*-R1 (5’- CGTTTTGATTTTCTGTTCTTATTACTTTATTA-3’).

**Southern blotting**

Mutants were verified by Southern blot using an intron specific probe. 2 μg genomic DNA were digested with HindIII (NEB) overnight. The blot was carried out using a DIG high prime labelling and detection kit (Roche) according to the manufacturer’s instructions.

**Western blotting**

Supernatants from 96 h cultures, grown anaerobically in TY, were concentrated eight-fold by chloroform-methanol-precipitation. Protein concentrations were standardized and run on Tricine gels 10-20% (Invitrogen) and transferred onto nitrocellulose membrane. The membranes were blocked with milk powder and then incubated with mouse monoclonal anti-TcdA antibody TTC8, mouse monoclonal anti-TcdB antibody 2CV (tgcBIOMICS) or immune-purified anti-Ia antibody specific for *C. perfringens* iota toxin component A (Ia), which cross-reacts with *C. difficile* CDTa [[2](#_ENREF_2)] respectively, followed by protein A-HRP (Sigma) or anti-rabbit-HRP (promega). The ECL Western blot detection kit from Amersham was used according to the manufacturer’s instructions.

**Cell toxicity assays**

The strains were grown in 8 ml of TY under anaerobic conditions as previously described [[1](#_ENREF_1)]. Samples were taken after 72 h, the optical density was measured and they were centrifuged and filtered. Supernatants were diluted in a four-fold series and 20 μl of dilutions were added onto monolayers of Vero and HT29 cells preincubated in 96 well plates for 48 h (at 37°C, 5% CO_2_). Cytotoxicity was recorded after 24 h as endpoint titres which were defined as the first dilution in a series for which HT29 or Vero cell morphology was indistinguishable from the negative controls (that is, cells which had been incubated with uninoculated *C. difficile* culture medium).

Vero and HT29 cells were grown in DMEM or McCoy's 5A, respectively, with 10% v/v foetal calf serum and 1% v/v penicillin-streptomycin at 37°C, 5% CO_2_ until confluent. Cells were detached using trypsin, and seeded into 96 well plates at a density of ca. 2 × 10^5^ cells/ml. All assays were carried out in triplicate. GraphPad Prism was used for statistical analysis. Significant differences were assessed using one way ANOVA tests followed by Dunnett’s multiple comparison tests.

**Analysis of faecal and caecal samples**

Faecal and caecum samples were plated on Fructose agar (*Clostridium difficile* agar base, Oxoid) with cycloserine cefoxitin, taurocholate and amphotericin to select for *Clostridium difficile*. The following primer sets were used to authenticate the various strain genotypes: primers CDSM0239 F1 (5’-GCTATTATTATGCCAGGATACTTTTATACACC-3’) and CDSM0239 R1 (5’- GACTCACTAATTTCTATTCCATATATTGATGC-3’) to confirm the cells were derived from *C. difficile* R20291, primers Cdi-tcdA-F2 and Cdi-tcdA-R2 [[1](#_ENREF_1)] to distinguish between the toxin A insertional mutants and wild type, the primers Cdi-tcdB-F1 and Cdi-tcdB-R1 [[1](#_ENREF_1)] to distinguish between the toxin B insertional mutants and wild type and the primers Cdi-027-*cdtA*-F1 (5’- TCAAGAGTTAATTAAACTAATATTGGGAGG-3’) and Cdi-027-*cdtA*-R1 (5’- CGTTTTGATTTTCTGTTCTTATTACTTTATTA-3’) to distinguish between the CDTa insertional mutants and wild type.

**Statistical analysis**

Statistical analysis was performed using GraphPad Prism. Data were analysed by 1-way-ANOVA followed by Dunnett’s multiple comparison test (cytotoxicity) or Student’s *t*-test (in vivo experiments). A statistically significant difference was considered to be p values of < 0.05.

**Figure legend S1**

**Figure S1. Characterisation of *C. difficile* toxin mutants.** **a**, Southern blot using an intron specific probe. The control plasmid (pMTL007C-E2) and genomic DNA of the wild type R20291 and 7 mutant strains was digested with HindIII, which resulted in a band of ca. 9 kb for the plasmid, 7.8 kb for the *tcdA*-mutation (in A^-^, A^-^C^-^, A^-^B^-^and A^-^B^-^ C^-^), just over 6.8 kb for the *tcdB*-mutation (in B^-^, A^-^B^-^, B^-^C^-^ and A^-^B^-^ C^-^) and ca. 4.7 kb for the *cdtA*-mutation (in C^-^, A^-^C^-^, B^-^C^-^ and A^-^B^-^ C^-^). **b and c**, Western blot probing culture supernatants with anti-TcdA-antibody (a) and anti-TcdB-antibody (b) (tgcBIOMICS). **d** Western blot probing for CDTa (provided by Prof. M. R. Popoff).

**References**

1. Kuehne SA, Cartman ST, Heap JT, Kelly ML, Cockayne A, Minton NP. The role of toxin A and toxin B in Clostridium difficile infection. Nature **2010**; 467:711-3.

2. Carter GP, Lyras D, Allen DL, et al. Binary toxin production in Clostridium difficile is regulated by CdtR, a LytTR family response regulator. J Bacteriol **2007**; 189:7290-301.


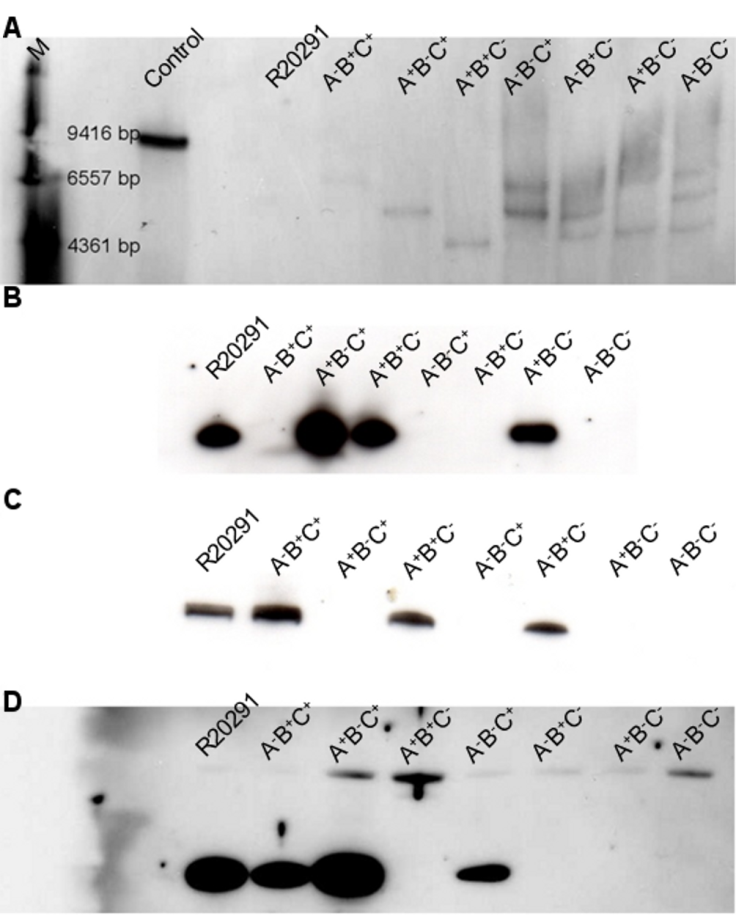

Supplement: Supplementary Data [file supp_jit426_jit426supp.docx]
